# Reproducibility analysis of bioimpedance-based self-developed live cell assays

**DOI:** 10.1038/s41598-024-67061-2

**Published:** 2024-07-16

**Authors:** Zoltan Vizvari, Nina Gyorfi, Gergo Maczko, Reka Varga, Rita Jakabfi-Csepregi, Zoltan Sari, Andras Furedi, Eszter Bajtai, Flora Vajda, Vladimir Tadic, Peter Odry, Zoltan Karadi, Attila Toth

**Affiliations:** 1 Department of Environmental Engineering, Faculty of Engineering and Information Technology, University of Pecs, Boszorkany str. 2, Pecs, 7624 Hungary; 2 Multidisciplinary Medical and Engineering Cellular Bioimpedance Research Group, Szentagothai Research Centre, University of Pecs, Ifjusag str. 20, Pecs, 7624 Hungary; 3 Symbolic Methods in Material Analysis and Tomography Research Group, Faculty of Engineering and Information Technology, University of Pecs, Boszorkany str. 6, Pecs, 7624 Hungary; 4 Institute of Physiology, Medical School, University of Pecs, Szigeti str. 12, Pecs, 7624 Hungary; 5 Department of Laboratory Medicine, Medical School, University of Pecs, Szigeti str. 12, Pecs, 7624 Hungary; 6 Department of Technical Informatics, Faculty of Engineering and Information Technology, University of Pecs, Boszorkany str. 6, Pecs, 7624 Hungary; 7 Institute of Molecular Life Sciences, HUN-REN Research Centre for Natural Sciences, Magyar tudosok korutja 2, Budapest, 1117 Hungary; 8 Semmelweis University Doctoral School, Ulloi str. 26, Budapest, 1085 Hungary; 9 Institute of Technical Physics and Materials Science, HUN-REN Centre for Energy Research, Konkoly-Thege Miklos ut 29-33, Budapest, 1121 Hungary; 10 John von Neumann Faculty of Informatics, Óbuda University, Becsi str. 96/B, Budapest, 1034 Hungary

**Keywords:** Assay systems, Biomedical engineering, Electrical and electronic engineering, Characterization and analytical techniques, Design, synthesis and processing, Sensors and biosensors, Electronic devices, Computational methods, Scientific data, Biological physics

## Abstract

Bioimpedance spectrum (BIS) measurements have a great future in in vitro experiments, meeting all the requirements for non-destructive and label-free methods. Nevertheless, a real basic research can provide the necessary milestones to achieve the success of the method. In this paper a self-developed technology-based approach for in vitro assays is proposed. Authors invented a special graphene-based measuring plate in order to assess the high sensitivity and reproducibility of introduced technique. The design of the self-produced BIS plates maximizes the detection capacity of qualitative changes in cell culture and it is robust against physical effects and artifacts. The plates do not influence the viability and proliferation, however the results are robust, stable and reproducible regardless of when and where the experiments are carried out. In this study, physiological saline concentrations, two cancer and stem cell lines were utilized. All the results were statistically tested and confirmed. The findings of the assays show, that the introduced BIS technology is appropriate to be used in vitro experiments with high efficacy. The experimental results demonstrate high correlation values across the replicates, and the model parameters suggested that the characteristic differences among the various cell lines can be detected using appropriate hypothesis tests.

## Introduction

In vitro testing, using primary and/or commercially available cell lines as experimental compounds of physiological or pathological processes, is indispensable and it is of distinguished significance for life sciences. Developing technologies which can increase the amount of information retrieved from these assays are in the focus of related research for decades^1,2^. Alterations of cell number, viability, cell death, morphology, gene expression and treatment-associated changes can be relatively easily detected, however, in most cases a fluorescent reagent, or a tag is also required. Thus, measuring several parameters simultaneously is further limited by the number of available fluorescent channels. In addition, it is also necessary to consider, that the introduction of fluorescent dyes and genetically encoded markers, or sensors into cells could affect the outcome of the experiment. The new type of tests on in vitro cell cultures, which are still ongoing, are very responsible for the processes taking place in the human body and the development of related diseases, with high precision and understanding.

In order to observe and examine these changes, it was necessary to develop new methods that are capable to determine functional properties of cells in real time, and in label-free and non-invasive ways, in contrast to currently broadly used techniques (such as, e.g., histological stainings), where the majority of them being very time-consuming and destructive for the samples. In this research, introducing reliable label-free monitoring of changes in biologically determined features would be an important step forward, however, this technology still has some serious limitations like requiring extraordinarily special equipment, finite options for multiplexing, rare availability, high price and low reproducibility as well^3^. Based on all these aspects, in vitro bioimpedance (BI) measurements seem to represent one of the most promising technical invention areas of scientific research, a real solution in terms of solving the problems arising in the previously mentioned tasks^3–5^.

Electrical resistance measurement, as the predecessor of BI measurements, goes back more than a hundred years^6^. The pioneers of the technique (Lord Kelvin, Frank Wenner and Conrad Schlumberger^6,7^) established the four-wire electrical resistivity measurement that is still used in geophysical measurements today^8^. Although DC excitation is preferred in these cases, the technological approaches discussed in this paper employ AC excitation. In contrast to most geophysical applications, the AC implementation of BI measurements adds significant complications in terms of both, instrumentation and modelling, since in this case crucial electrochemical effects are considered that are not present in DC measurements^7^.

Novel techniques in the field of BI hold the potential to introduce groundbreaking advancements, such as the development of a completely non-invasive and label-free tomograph or spectrometer. These devices enable researchers to swiftly and inconspicuously gather an extensive range of real-time measurements during in vivo experiments with the flexibility to operate at any time of the day^9^. To exploit the potential of this nowadays’ new technique, many technological barriers still need to be eliminated^10–12^. As far as difficulties emerging with the employment of the technique are concerned, a further, almost impenetrable problem is that the effects of the measurement artifacts also depend on the unknown impedance itself^13^. A thorough review of the international literature revealed, that the measurement methodology and the mathematical methods generally implemented in the data evaluation procedure, as well as other efforts to improve BI-based technologies have not fulfilled the expectations yet^11,14^.

Bioimpedance spectroscopy (BIS) has become a widely used technique, since the 1980s^15^ for the assessment of human body composition, with special emphasis on determination of water content^16–19^ and also for differentiation of cancer cells from the normal ones^20^. Since the technique is considered as non-invasive, it can be repeated virtually indefinitely, with no adverse consequences. In spite of successful employment of non-commercial equipment in several previous cases, only the commercial products, which functioning was based on the original methodological elements, have been able to become the gold standard for measuring total body water in the clinical practice^11^. One of the possible reasons, why BIS did not conquer as much use as possible in in vitro experiments, is the fundamental structure of such devices^10,15^. Commonly used BIS systems utilize only two-electrodes (current injection and voltage measurement) to analyze the biological samples^3,11,21^. While this set-up is suitable for some specific tasks, it is greatly reducing the specificity of the obtained results due to the contact impedance problem^22^ referring to the yet, partly unknown factors that influence the connection between the living cells and the conducting electrodes^21^.

The most widely used and well known BI based, label-free in vitro approach is the electric cell-substrate impedance sensing (ECIS) method, that was published first by Giaever and Keese in 1984^23–25^. During the ECIS measurement, the cells growing directly on the surface of the coplanar thin layer electrode located at the bottom of the cell culture vessel^21^. Therefore, the material of the electrodes plays a key role in the implementation of ECIS, since in the case of the two-electrode systems, the cells attach to these electrodes and the electrode, electrolyte, cell interface is developed^23^. Obviously, this means, that only biocompatible and non-toxic materials are used as the electrodes. According the literature, ECIS implementations using electrode materials such as gold, platinum, palladium, titanium, indium tin oxide (ITO), or other organic electrode materials such as 3,4-ethylenedioxythiophene, polystyrene sulfonate, polyaniline and graphene oxide^26,27^. All these materials are preferred due to their biocompatibility, stable electrochemical properties, optical transparency (ITO) or compatibility with printing processes^26,27^. In the ECIS standard, a thin layer of gold film is used as an electrode material, because it is easy to create it by lithography for each application area^26^. Additional benefits of the gold film electrode are its high conductivity and the almost ideal capacitive behavior (after a cystein treatment)^28,29^. In recent years, however, graphene has also emerged as a potential electrode material, mainly due to its advantageous properties of being highly flexible (even manually can be applied to electrodes), as well as its advantageous multi-frequency behavior^26,30^. The two-electrode method causes the electrical double layer, that limits the frequency range of the method in the low frequency region (< 100 Hz)^26^. In many cases, graphene based solutions allow the frequency range of the two-electrode measurements to be extended to lower frequencies^26,30^.

In the literature, for a few in vitro BIS studies, the recorded spectra are evaluated using a Cole–Cole model^31^, and the detection of biological processes is obtained by changes in the model parameters. During the impedance studies of healthy and tumor cells of rat liver epithelial cell lines (WB-F344 and WB-ras), clear differences in the Cole–Cole parameters were observed^32,33^. Hays et al.^34^ performed a high frequency range (500 MHz to 40 GHz with a 9.897 MHz resolution) glucose detection study on blood plasma samples obtained from Sprague Dawley rats. The Cole–Cole model was used to evaluate the in vitro results. In addition to this, Wang et al.^35^ presented an in vitro study of human lung tissue using the modeling of the dielectric with the Cole–Cole model (100 Hz and 100 MHz range).

One of the cornerstones of the in vitro BIS measurements is the reproducibility, which is very difficult to define in precise numerical terms for this technology. However, precise knowledge of the variations characteristics, resulting from the repetition of measurements, is not only of utmost importance from both, the biological and measurement technical points of view, than it also greatly influences the obtained results. In addition, it has a huge economic impact on biological research^1,2,36^. The literature is generally positive about the reproducibility of BIS measurements, especially for clinical body composition studies^37–39^. For BIS techniques used in in vitro experiments, researchers generally consider the reproducibility of the method to be sufficient^4,40^. Considering the majority in vitro BIS methods, ECIS is perhaps the most advanced and sophisticated technique. This method, using two-electrodes and usually a discrete frequency, has attracted the attention of researchers because its wide range of applications, including reproducibility testing^3,41^. Gelsinger et al.^21^ detailed how the cell identification methodology they have invented could help to move biological science out of the “reproducibility crisis”. Atienzar et al.^42^ introduced an assay by using statistical methods (mainly correlation calculations). The reproducibility of their system with different coatings and a human hepatoma cell lines (HepG2) was tested. Their real-time cell analyzer also works on the ECIS principle^42^. This work described, that the achieved results represent the first systematic, objective, statistically validated series of experiments specifically performed for bioimpedance-based in vitro biological methodology^42^.

Previously, results enabling the implementation of BI methods on a new technological basis have been achieved^43–45^. The authors had introduced and validated a self-developed BIS system and approach to strikingly increase reproducibility and precision of the measurements using a current circuit model^12^. Vizvari et al.^12,46–48^ have achieved outstanding results in improving the signal-to-noise (SNR) ratio of BI measurements in lower frequency ranges (from DC to hundred kHz), and they have eliminated the effects of the measurement artifacts in the whole range. This unique technique is based on the standard four-electrode method^11^, which allows to eliminate the contact impedance between the electrode surface and the investigated material^12^. For this reason, Vizvari et al.^12^ demonstrated the outstanding properties of their BIS technique, where they successfully validated a digital lock-in amplifier prototype developed for the use in the implementation of this technology. The precision of state-of-the-art voltage comparison technique and the self-developed technique is compared in a dedicated study^46^. For this, special RC circuits built from ultra-precise resistors were applied (temperature coefficient is less than 2ppm/°C which is a part per million per Celsius)^46^. The results demonstrated that the technological implementation was able to detect impedance values with a variance equal to the thermal coefficient value (2 ppm)^46^. In fact, in some cases even lower variance values were detected (< 2 ppm), while the variance of the conventional measurement solution was significantly higher (5–33 ppm)^46^. These results indicated that the self-developed measurement technique and the associated prototype are able to achieve in vitro BI measurements, whose variability is generated by the in vitro measurement setup and the biological processes followed and not by the technological background. Hence, the variance of the cell culture-free set-up and the variance of the whole system with cell culture are presented separately in this paper.

The self-developed approach for in vitro BIS measurements will allow the detection of special material quality information in the frequency domain. For this purpose, it was necessary to develop measuring plates that are adapted to the advantageous properties of the specific four-electrode technology, while minimizing the possibility of additional errors. The basis of the self-developed live cell assays are the BIS plates invented by authors^48^. In this case, the primary objective of researchers is to maximize the suppression of the effects of electrode-cell, electrode-electrolyte transition, that originates from the excitation and ground electrodes on which the ECIS technology is based on. To increase the rejection effect, the excitation electrode surfaces have been minimized and ignored from BIS data recordings. As a consequence, the surface area of the sensing electrodes has been maximized to achieve the highest possible sensitivity, which specifically detects structural changes in the cells grown on the sensing electrodes. The inputs of the measuring device are connected to the plates using silver wires, and the cell cultures are grown in two prepared cell-culture dishes (Petri dishes) on plates, which contain electrode arrangements aligned to the measurement. Therefore, the hand-made measuring plates represent a new extension of the in vitro BIS technology. The blueprint and the whole fabrication procedure of the actual BIS plates, is described in the Supplementary Methods online (Section S1.1).

Further, to highlight the benefits of the applicability of the deployed technology, the validation and the usage of the measurement plates are described in this study. The applied plates are produced and validated at completely different dates within a time frame of almost one year. A subset of the validated assays is used for viability testing to verify biocompatibility, while the other subset is applied for validation and in vitro BIS analysis of three different cell lines (two tumor and one mesenchymal stem cell lines) which replicates to investigate the reproducibility of the method. To evaluate the results of the BIS analysis on cell lines, a standard single-dispersion Cole–Cole model^31^ is used, and by extracting the model parameters, the possibility is gained to compare the cell lines based on the model.

In order to demonstrate the beneficial properties of the proposed technology and the BIS data, correlation and normality tests are performed. The variance and the significance of the extracted model parameter sets of the individual cell lines are included in the study too. Considering all these outcomes, authors are devoted to gain a special invention to the technological achievements of ECIS, a completely proprietary in vitro BIS approach, that is able to identify structural changes in cell cultures in a reliable, non-destructive way.

The paper is organized as follows. The first section is the introduction, followed by the materials and methods section. The next section focuses on results and discussion. The conclusions and the future plans are described in the last section.

## Materials and methods

### Self-produced live cell assays

The plates are based on a 0.5 mm thick, transparent polycarbonate sheet (Lexan Clear Polycarbonate 500 Micron Thick Film Sheet). The graphene is chosen for the electrodes, since it is more efficient than the gold from a measurement and biological point of view. The surfaces filled with black color are the electrodes in Supplementary Fig. S1, which are made with graphene paint (SunChemical C2171023D1 Carbon Graphene Paste diluted 1:1 with butyl acetate). A silver wire (0.5 mm, with 99% purity) was applied to the graphene painted surface. The silver wires, cut at 5 cm are fixed with a two-component epoxy resin-based adhesive. Bottom-open Petri dishes for measuring cell cultures are placed on the polycarbonate sheets. Petri dishes (with a diameter of 35 mm) are fixed to the polycarbonate plates with a non-toxic silicone-based adhesive. The polycarbonate sheets are cut to nearly same size (115x48 mm) to fit the dimensions of the guide surface, silver wires and Petri dishes. Prior to the application of the graphene paste, the polycarbonate sheet is degreased and disinfected (with ethanol and i-propanol based disinfectant, 74% in total). The electrodes are formed by hand-coating with two to three layers of graphene paste to ensure the complete coverage. Before applying the new layer of graphene paste, the plates are completely dried. Once the graphene hand coating is finished, the Petri dishes are fixed to the polycarbonate sheet using silicone-based adhesive. The silver wire is cut into 5 cm pieces and straightened to fit tightly to the graphene painted conductive surface. The silver is coated with graphene at the contact surface of the graphene and silver. The silver is temporarily fixed with adhesive tape and then permanently fixed with a two-component epoxy resin-based adhesive after drying. The unattached end of the silver wire is bent at nearly 90° to allow easy attachment of the cables to the electrodes during the measurement.

The in vitro results are obtained in several experiments with replicates separated in time and space (not at the same time, not at the same place). The sequence numbers of the plates used in in vitro investigations and the dates of the experiments described above are summarized in Table 1. The plates are prepared at different time points and validated with physiological saline at different time points. Each of the three cell types is plated in four Petri dishes, hence that each experiment is well separated in space and time (Table 1).

**Table 1:** Summary of the producement and validation measurement dates of the plates used in this paper.

Plate no.	Production date	Side no.	Validation date	Meas. date	Preparation
2022-04-27-01	2022-04-27	Side 1	2022-05-25	2022-06-08	MCF7
		Side 2	2022-05-25	2022-06-08	231
2022-04-27-04	2022-04-27	Side 1	2022-05-25	2022-06-08	MCF7
		Side 2	2022-05-23	2022-06-22	MCF7
2022-05-02-04	2022-05-02	Side 1	2022-05-25	2022-06-08	231
		Side 2	2022-05-23	2022-05-27	MCF7
2022-05-03-08	2022-05-03	Side 1	2022-05-24	2022-06-22	231
		Side 2	2022-05-24	2022-05-27	MSC
2022-05-03-09	2022-05-03	Side 1	2022-05-24	2022-06-08	MSC
		Side 2	2022-05-24	2022-05-27	MSC
2022-06-14-02	2022-06-14	Side 1	2022-06-17	2022-06-22	231
		Side 2	2022-06-17	2022-06-08	MSC
2022-08-17-09	2022-08-17	Side 1	2022-08-30	2022-09-28	MCF7 med
		Side 2	2022-08-30	2022-09-28	231 med
2022-08-17-13	2022-08-17	Side 1	2022-08-31	2022-09-28	MCF7 med
		Side 2	2022-08-31	2022-09-28	MSC med
2022-08-17-14	2022-08-17	Side 1	2022-08-31	2022-09-28	231 med
		Side 2	2022-08-31	2022-09-28	MSC med

### The BIS measurement technique

**Figure 1:**
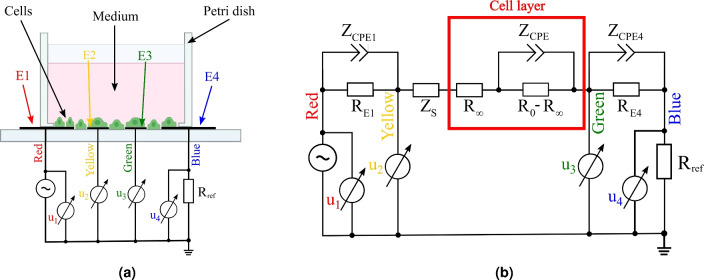
Schematic drawings (not to scale) representing the application of the self-developed in vitro four-electrode method for BIS measurements in case of the self-produced graphene plates (**a**) and the equivalent circuit model (**b**) (drawings not to scale).

The BIS measurements are performed using a self-developed, modified four-electrode technique introduced by Vizvari et al.^12^. The idea behind this technique is that errors caused by excitation electrodes and residual impedances are suppressed simultaneously. The common-mode rejection of these error phenomena is based on connecting the generator directly to one electrode and the ground to the other through a resistor^46^. This effectively shifts the potential values by a constant value. The compensation with the constant and the division during the impedance calculation are done digitally, which eliminates the errors of the symmetrically designed measuring circuit^12^. The self-developed BI measurement technique (which forms a basis for BIS and for tomographic measurements) is currently patented in several countries around the world^43–45^. The principle of the self-developed in vitro technique is illustrated in Fig. 1.

In the previous publications, Vizvari et al.^12,46,47^ have shown that the application of this data acquisition and evaluation method can increase the efficiency and accuracy of EIS measurements even over a wide frequency range compared to other techniques. As shown in Fig. 1, a resistor ($R_{ref}$) is connected in series with the measuring cell, which shifts the voltage across the cell by a constant value. Therefore, the excitation electrodes (E1 and E4 in Fig. 1a) are at non-zero potentials, thus the electrical double layer is developed on the both of the electrodes^40^. Although the ground point is not directly connected to the biological structure under the investigation, the current flowing between the voltage generator (E1) and electrode E4 is the result of the potential difference carried by the generator. In Fig. 1b, the electrical double layers occurring at electrodes E1 and E4 is represented by the simplified Randles cells consisting of equivalent circuit elements $Z_{CPE1}$, $R_{E1}$, $Z_{CPE4}$, $R_{E4}$ and $Z_S$, respectively^40^. The impedance $Z_S$ symbolizes the impedance of the solution placed in the measuring cell, while the impedance of the Constant Phase Elements (CPEs, $Z_{CPE1}$ and $Z_{CPE4}$) includes the capacitance arising at the electrodes. Exploiting the fact that the effect of the electric double layer is in the area close to the electrode (since the charge distribution is maximum at the excitation electrodes and decreases exponentially away from them^40^), the E2 and E3 measuring electrodes are placed in separate locations (Fig. 1a). In the circuit shown in Fig. 1b, this is illustrated by placing the E2 (Yellow) and E3 (Green) measurement points within the equivalent circuit, independently of the generator.

**Figure 2:**
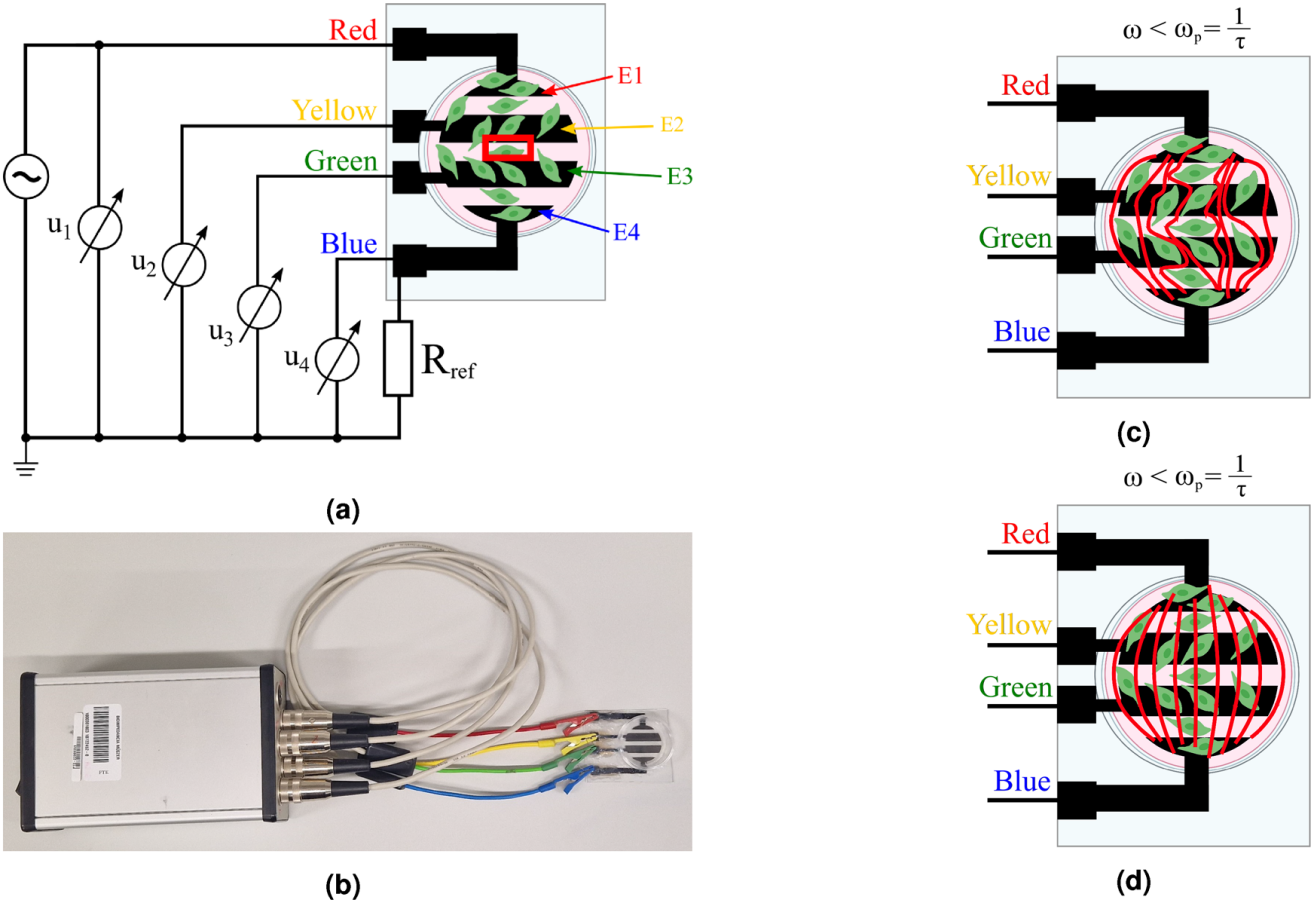
Schematic drawings (not to scale) representing the self-developed BIS principle [(**a**) the measuring principle, where the red rectangle depicts the approximate region where the live-cell microscopy was performed, (**b**) an assembled device, (**c**) the electric current lines at low frequency ($\omega < \omega _p$), (**d**) the electric current lines at high frequency ($\omega > \omega _p$)].

The impedance of the cell layer (the red rectangle in Fig. 1b) can be understood on the basis of the following facts summarized in Fig. 2.

According to the measurement principle in Fig. 2a, if the excitation frequency ($\omega$) is lower than the natural frequency of the cell layer ($\omega _p$), the cell membranes act as insulators, hence the electric current flows only in the extracellular space (Fig. 2c)^40^. The resistors in the red rectangle in Fig. 1b are then connected in series with the impedance of the medium, since the impedance of the CPE is infinite. In this case, the resultant of the resistance of the extracellular space ($R_0$) and the impedance of the medium ($Z_S$) can be measured. With increasing the frequency, the cell membranes become conductive and the electric field is established inside the cells^40^. Fig. 2d illustrates, that the current lines also pass inside the cells, so the resistance $R_0 - R_{infty}$ shown in Fig. 1b is shunted by the impedance $Z_{CPE}$, and therefore the resultant of the resistance $R_{\infty }$, which represents both the extracellular and intracellular space, and the impedance of the medium is measured. Obviously, the cell membrane is not considered as ideal capacitor, hence it is modeled with CPE^40^.

Naturally, the cells grow on the entire surface of the Petri dish, hence there are also cells on the surface of electrodes E1 and E2. At these excitation electrodes, the electrode-electrolyte-cell interaction (on which the ECIS measurement is based^23^) is formed, thus the simplified Randles element elements shown in Fig. 1b are also affected. More importantly, the two-electrode measurements obtained from this yield time-varying impedance BIS data. However, these effects are neglected in further studies and the primary focus is on the ability of the four-electrode method to suppress all these effects.

The spectrum of the cell culture $Z_{cell}$ is calculated at each frequency point using the potential values $u_2$, $u_3$ and $u_4$ according to the following formula:

1 $$\begin{aligned} Z_{cell} = R_{ref} \frac{u_2 - u_3}{u_4} \end{aligned}$$

The efficiency of the method is based on the fact, that during the data acquisition, common mode suppression is implemented to ignore measurement errors and artifacts^12^. The measurement principles illustrated in Fig. 1 and in Fig. 2 provide the possibility to calculate the spectra of two-electrodes of the measuring cells (between E1 and E4) from the collected data. The method is introduced in the Supplementary Section S1.2.

The measurement system used is a high-precision digital lock-in amplifier developed by Vizvari et al.^12,46,47^, using voltage generator excitation specifically for measuring small electrical signals. The main features of the system are:

- maximum excitation voltage is 10 V peak-to-peak (the range of excitation is 110 dB),
- maximum excitation current 1 mA,
- measurement frequency range 1 mHz–100 kHz (with 0.01 Hz resolution),
- the four-channel instrument execute the lock-in algorithm in the IEEE.754 64-bit double precision domain^12^,
- the accuracy of the measured data is less than 1 ppm for amplitudes and for phase less than 0.01°,
- it is battery-powered (maximum 6.5 h autonomous operation time).

An assembled instrument is shown in the Fig. 2b.

### Physiological saline measurements

The validation measurements of the handmade plates are performed with a physiological saline solution series. A series of 100%, 75%, 50%, 25% and 0% solutions are prepared using physiological saline and deionized water. The 100% solution corresponds to the full physiological saline solution. During the measurements, a 300 $\mu l$ of solution from each of the five concentrations is pipetted into the actual Petri dish and the BIS measurements are started and repeated five times in succession. The measurements were performed with specific settings: 40 dB generator signal attenuation (100 mV peak to peak), frequency sweep frequency range 0.1 Hz–100 kHz at 5 points per decade (thus a spectrum is recorded at 30 frequency points, which takes approximately 90 s in time). All the BIS measurements are repeated 5 times in the case of one Petri dish.

### Medium control plates

It was interesting to study, how the proprietary BIS technology can separate prepared Petri dishes containing cells from the ones without cells. For this purposes, 3 of the 9 plates (2022-08-17-09, 2022-08-17-13, 2022-08-17-14) are prepared specifically to study the effect of the extracellular matrix without live cells and the medium mixture on the measurement results. These plates are prepared with only the coating and the corresponding media (Table 1). These will be referred as medium controls in the followings. In this case, a comparison is made between the 100% saline solution, prepared Petri dishes without cells and the prepared Petri dishes with cells. In this way, it can be concluded, is the introduced method able to detect in vitro cell cultures.

### Cell-lines and the coating

The human breast cancer cells lines (MCF7, MDA-MB-231, hereinafter referred to as 231) were obtained from the National Cancer Institute’s Developmental Therapeutics Program (National Institutes of Health). The adipo-derived mesenchymal stem cells (Ad-MSC, hereinafter referred to as MSC) were isolated from a liposuction sample taken from a 30-year-old healthy female donor. The cell line was characterized according to the International Society for Cell & Gene Therapy standards and described by Vajda et al.^49^.

MCF7 and MDA-MB-231 cell lines were cultured in Roswell Park Memorial Institute (RPMI) medium, supplemented with 10% fetal bovine serum (FBS, Gibco, Waltham, Massachusetts, USA), 5 mmol/L l-glutamine and 50 unit/mL penicillin and streptomycin. MSCs were cultured in the 1:1 mixture of Dulbecco’s modified Eagle’s medium and F12 Nutrient Mix (DMEM-F12, Gibco) supplemented with 10% fetal bovine serum (FBS), 1% l-glutamine, 0.1% gentamicin, and 16 ng/mL fibroblast growth factor 2 (Peprotech, London, UK). Cell culture reagents were obtained from Thermo Fisher Scientific, Massachusetts, USA. All cell lines were cultured at 37 °C with 5% $CO_2$. For the coating of the BIS plates the 0.5 mg/mL fibronectin solution (Sigma-Aldrich, St. Louis, MO, USA) was diluted with sterile distilled water in 1:100 ratio. 1 mL fibronectin solution was added per well and incubated for 1.5 h. After the incubation, the fibronectin solution was discarded, and the wells were washed with PBS. Finally, the coated plates were disinfected with UV light for 45 minutes before the cells were plated.

For each cell culture, the BIS data was recorded after 24 hours of incubation from cell seeding.

### Cell viability assay

The purpose of the viability tests is to verify the biocompatibility of the introduced in vitro BIS technology. For viability assays, 9 (3 for each cell lines) separated pieces of BIS plates were chosen (fabricated using the same methodology described in Section “Self-produced live cell assays”), which are prepared and seeded with the same methodology described in Section “Cell-lines and the coating”. In order to establish this test, $1 \times 10^5$ cells are seeded into each well and the viability of the cells is measured at 3 time points: 24h, 48h and 72h after the cells were plated. The viability is assessed using the PrestoBlue assay (Invitrogen, Waltham, MA, USA). The PrestoBlue™ Cell Viability Reagent is diluted with culture medium to 5%. The medium is discarded from the BIS plate wells and 1 mL of 5% PrestoBlue™ solution is added per well. Plates are incubated for 1.5 h at 37 °C/5% $CO_2$. After the incubation, a three 100 µL solution is pipetted to 96 well plates from each well and the fluorescent signal is measured using an EnSpire microplate reader (Perkin Elmer, Waltham, MA, USA). The BIS measurements are performed on each plate on the side 1 in 24 h time point, while there is no measurement on the side 2. Following the BIS measurement taken in 24 h, the PrestoBlue™ solution is discarded from the BIS plates and the medium is renewed. The procedure is repeated daily for 3 days.

### Live cell imaging

JuLI™ Stage Cell History Recorder (NanoEnTek, Seoul, Korea) is used to monitor the proliferative capacity of the cells on the BIS plates. $1 \times 10^5$ cells are plated into each well and images are taken every 4 h for 72 h, at 10 × magnification. The data are evaluated using the JuLI™ STAT (v. 2.1.0.5, www.julistage.com) and GraphPad Prism 8 (v. 8.0.0, San Diego, California USA, www.graphpad.com) software. The images included in the manuscript were mostly taken between the E2 and E3 electrodes, close to the middle of the well. Fig. 2a is extended with a red square, approximately showing the location of the field of view used in live-cell microscopy.

### Data evaluation and analysis

#### Graphical representations

During the measurements, the impedance of the cell culture $Z_{cell}$ spectrum data is calculated from the recorded raw data based on the considerations described in Vizvari et al.^12^.

For the physiological saline validation measurements, a total of 450 impedance spectra data were recorded by performing 9 plots, 18 Petri dishes and 5 measurements per concentration of saline, or 5 measurements per concentration. During the further measurements (cell culture and medium control), the setup of the measurement system (Fig. 2b) is practically identical to the setup described in Section “Physiological saline measurements”. During each data recording taken in the Petri dish, 5 impedance data were recorded. In each of the 9 plates, and 18 Petri dishes 5 measurements were performed, i.e. a total of 90 BIS data were measured during the experiment.

Next, the mean and the standard deviation of the spectra of 100% solution, medium control and the spectra measured on the corresponding cells (but containing the same medium) are plotted in the same graph for the 6 medium control Petri dishes (Section “Medium control plates”). For the 100% physiological saline and the medium control data, only the results measured on the same plates (Section “Medium control plates”) are considered in the evaluation.

Afterwards, for the remaining 6 plots where 3 different cell cultures are distributed, the recorded $Z_{cell}$ spectra are grouped by cell type and the groups are plotted as described in previous.

#### The Cole–Cole model

For the evaluation of the spectra measured on cell cultures, a model-based evaluation is preferred to allow the dielectric properties of biological structures to be properly studied in vitro^50^. For this purpose, the Cole–Cole model^31^ is applied, which describes the frequency domain behavior of the biological structures. The Cole–Cole equation describing the equivalent circuit is expressed as follows:

2 $$\begin{aligned} Z(j\omega ) = R_{\infty } + \frac{R_0-R_\infty }{1+(j \omega \tau )^\alpha }, \end{aligned}$$

where $Z(j \omega )$ is the complex impedance, $R_{\infty }$ is the resistance corresponding to the $\infty$ frequency, $R_0$ is the resistance corresponding to 0 Hz frequency, $\tau$ is the time constant, $\alpha$ is the exponent parameter ($0 < \alpha \le 1$), $\omega$ is the angular frequency, and $j = \sqrt{-1}$.

The biological information provided by the Cole–Cole parameters ( not different from the existing practice) are the follows. Considering that the membrane of the cells is insulating at low frequencies, the parameter $R_0$ refers to the resistance of the extracellular space and the membrane integrity of each cell^32^. The characteristic time constant $\tau$, together with $R_0$, indicates the membrane capacity $C_m$ of the developing cell layer^32^. The $\alpha$ parameter is correlated with extracellular space and cell size distribution^32^. While $R_{\infty }$ describes the resistance of the extracellular and intracellular space (based on the fact that at high frequency the cell membrane becomes conducive)^32^.

The extraction of the Cole–Cole model parameters ($R_0$, $R_{\infty }$, $\tau$, $\alpha$) is performed using a simple non-linear Levenberg–Marquardt based regression algorithm in MATLAB. The fit of the Cole–Cole function is performed directly on the magnitude vs. frequency data for each cell culture measurement separately.

The goodness of fit is measured using the following formula^12^:

3 $$\begin{aligned} R^2 = 1 - \frac{\sum _{i = 1}^{n} (\hat{Z}_{cell_i} - Z_{cell_i})^2}{\sum _{i = 1}^{n} (\overline{Z}_{cell} - Z_{cell_i})^2}, \end{aligned}$$

where $Z_{cell_i}$ is the *i*th data point in $Z_{cell}$ vector, $\hat{Z}_{cell_i}$ is the *i*th data point calculated from the extracted Cole–Cole parameters (corresponding to the frequency points of the actual measurement), $\overline{Z}_{cell}$ is the average of the $Z_{cell}$ vector, and *n* is the number of data points in $Z_{cell}$.

#### Statistical evaluation

In the first step of statistical evaluation, correlation values are calculated using the Pearson correlation coefficient^51^. The correlation values are obtained for repeated measurements in the case of the saline measurements and for the all three cell lines, independently from the time and place of the plate production.

Since the Cole–Cole model^31^ is specifically designed to characterize biological structures, and since the physiological saline solutions do not contain cells, data processing for these data is based on isolating the frequency value measured at the median value of the measurement frequency range (160 Hz) for each spectrum and using this value for the further statistical analyses. The 450 spectral values are sorted into 5 concentration groups and, after the standardization, normality test are performed using one-sided Kolmogorov–Smirnov test^51^. Moreover, for measuring the selectivity of the method, two-sided Kolmogorov–Smirnov test^51^ is used to determine whether the parameter groups for each concentration, or cell lines, are significantly differ from each other.

For the plates containing cell cultures, the calculated Cole–Cole parameter values are sorted by cell types, then the normality of each parameter group is tested using the one-sided Kolmogorov–Smirnov test^51^ in the same way as in the case of the evaluation of the saline solution measurements.

In the case of the model parameter sets, the results of the significance test are plotted on bar charts. The mean of the data sets represents the height of the columns, and the markers above the lines connecting the compared columns indicate the level of significance of the difference between the groups. The standard deviation of the parameter sets in each column is also depicted in these figures. The significance notations, based on the p-values, are the followings: n.s. denotes non-significant, * indicates 0.05, ** indicates 0.01, *** means 0.001, > *** means 0.0001 significance level.

A significance level of 0.01 was used for all statistical tests. All the steps of data processing, data evaluation and statistics were performed in MATLAB environment.

### Ethics declarations

The adipose tissue derived mesenchymal stem cells were retrieved from a liposuction sample taken from a 30-year-old healthy female donor and processed with ethical permission from the Hungarian Medical Research Council. The cell line was established in 2012 in the Institute of Enzymology of the Hungarian Academy of Sciences by Tátrai et al. and was published in the same year (Tátrai et al.: Combined introduction of Bmi-1 and hTERT immortalizes human adipose tissue-derived stromal cells with low risk of transformation, Biochemical and Biophysical Research Communications, 2012; https://doi.org/10.1016/j.bbrc.2012.04.088). The study was conducted in accordance with the Declaration of Helsinki, and approved by the Ethical Committee of the Hungarian Medical Research Council (ETT; ID: 24083-3/2013/HER). This work mainly focusing on technological development without using any biological and/or tissue sample. The low number of validating in vitro experiments using only cell lines established decades ago. No human experiments were involved in our study.

## Results and discussion

### Viability results

**Figure 3:**
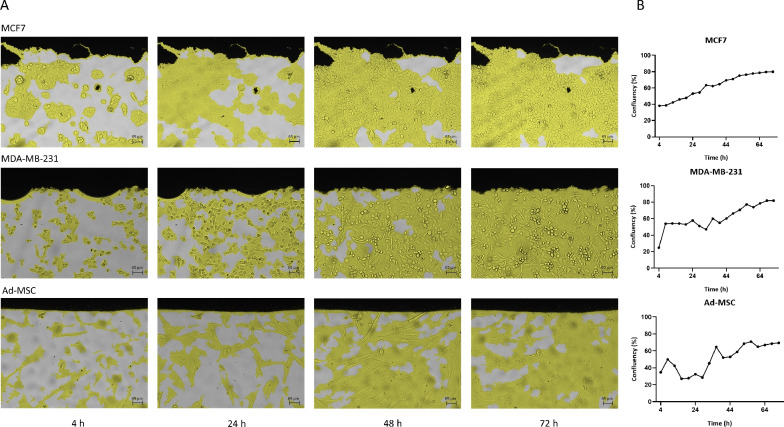
Viability and proliferative capacity of the cells on BIS plates [(**A**) video microscopy, (**B**) quantitative representation of the changes in confluence, based on the images presented in (**A**)].

The results of viability tests are summarized in the Fig. 3. Areas, marked with a red rectangle in Fig. 2a, of the BIS plates were carefully chosen to ensure, that the graphene-covered surface regions will remain within the field of view. Additionally, the approximate field of view is highlighted on the Fig. 2a. As it shown in Fig. 3A, cells were marked in yellow by the JuLI™ STAT program, allowing the monitoring of the changes in confluency. The number of cells were both visibly (Fig. 3A), and quantitatively (Fig. 3B) increased suggesting undisturbed proliferation and indicating that the graphene surface of the plates did not affect the viability of the different cell lines. While there are some outlier confluency data points on Ad-MSC’s growth curve around 24 hours, these are the results of the special phenotype of mesenchymal cells. Ad-MSCs are non-cancerous and originates from a fundamentally different tissue (mesenchyme, or connective tissue), than the other two epithelial cancer cell lines. Therefore, their proliferation rate, size and morphology is different and quickly changes over time depending on the microenvironment and motility. These changes decrease the accuracy of cell detection and confluency measurement by the software.

**Figure 4:**
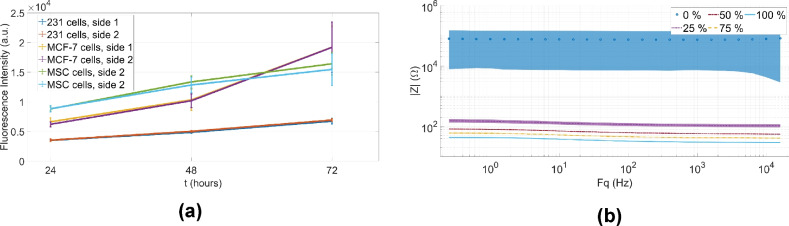
Summary of viability results (**a**) and the spectra measured on physiological saline solutions (**b**). (**a**) The impact of BIS measurements on the proliferative capacity of cells (Comparison of side 1 (BIS measured cells) and side 2 (no measurement) of the BIS plates.) (**b**) Summary of the physiological saline validation measurements (bold and/or dotted lines indicate the average spectra, while the bands in different colors corresponds to the standard deviation).

The two-sided Kolmogorov–Smirnov significance test is used to test whether the difference between the sides 1 and 2 is significant. The results are summarized in Supplementary Table S1 online. As it is shown in Fig. 4a, no difference is observed between the two sides in terms of fluorescence intensity, indicating that the viability and the proliferative capacity of the cells is not affected by the BIS measurement.

### Plate validation results

In the first step, spectra recorded during the tests are plotted in Fig. 4b, which indicates, that the recorded spectra from the 9 plates are very similar. The width of the bands around the averages seem to decrease with increasing the concentration. The shapes of the spectral curves are very similar, there is no overlap between the groups of curves measured for different concentrations, they are well separated from each other in each group. Apparently, the average curves are parallel, since the changing of physical concentration results in a simple shift, however no significant change in curve shape is observed. For a more detailed analysis, let consider the following results.

Based on the Section 2.8.3, the range of Pearson correlation values are between 0.93 and 1, in most cases the typical correlation value is around 0.99 for the repeated BIS measurements.

The normality test results of the impedance values measured at 160 Hz for each saline concentration are summarized in Supplementary Table S2 online. Considering the normality test results of the impedance values measured at 160 Hz (Section “Physiological saline measurements”), the data have a standard normal distribution in all cases.

### Medium control plates and cell line BIS results

**Figure 5:**
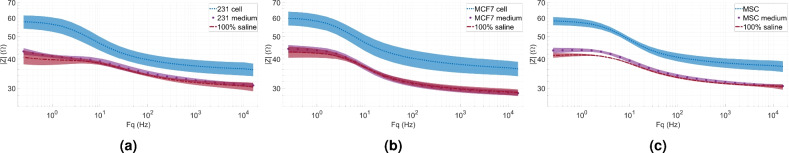
Comparison of the 100 % saline, medium controll and the corresponding in vitro BIS data in the case of 231 (**a**), MCF7 (**b**) and MSC (**c**) cell lines prepared based on the instructions in Section “Cell-lines and the coating” (100% solutions and medium control results are only considered in the same Petri dishes; bold lines represent the average spectrum, while the coloured bars represent the standard deviation).

After the analysis of the saline validation results, the medium control and cell line plates are compared based on the methodology described in Section “Medium control plates”. The corresponding BIS data are plotted in Fig. 5.

Based on Fig. 5, it can be generally observed, that the spectra of the 100% solution and the medium controls (placed in the same Petri dishes) are very similar, with a lot of overlaps between the curves. However, based on the cell culture BIS data, it can be clearly seen, that all three spectra (Fig. 5a for 231, Fig. 5b for MCF7 and Fig. 5c for MSC cells) are very distinct from the other two BIS data sets. Furthermore, the plots of the cell culture data are clearly distinguishable from the spectra measured on non-cell cultures. Based on these results, the graphical analysis clearly shows a complete separation between the cell culture curves and the non-cell culture results. Moreover, the width of the standard deviation intervals for BIS measurements performed on the cell lines does not change depending on the frequency.

The calculation of the two-electrode spectra (based on the equation is Supplementary Section S1.2) allowed by the measurement principle (Figs. 1 and 2) is performed by comparing the 100% saline, the medium control and the cell lines. The results are presented in Supplementary Section S2.1.

### Cell culture BIS results

**Figure 6:**
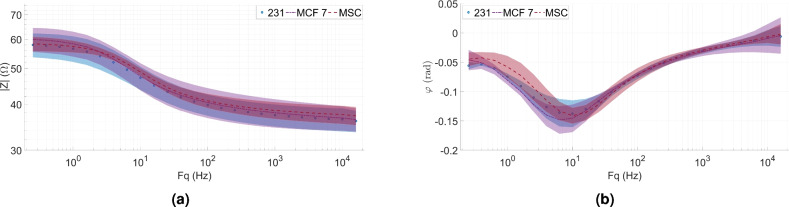
The magnitude (**a**) and the phase (**b**) of BIS data recorded on the cell culture cell lines prepared based on the instructions in Section “Cell-lines and the coating” (bold lines represent the average spectra, while the width of the bands in different colours corresponds to the standard deviation).

A summary of the spectra measured on the remaining 6 BIS plates are shown in Fig. 6. Based on Fig. 6, a lot of overlaps can be observed between the data sets. These considerations are noticed in the case of magnitude (Fig. 6a) and phase (Fig. 6b) plots respectively. Due to the limitations of the graphical analysis, it is considered important to perform further statistical analyses of the cell line BIS data and the extracted Cole–Cole parameters. The results of the cell culture BIS measurements are also illustrated in a Nyquist plot in the Supplementary Section S2.2 (Supplementary Fig. S4).

Similarly to the correlation analysis of the saline data in Section “Physiological saline measurements”, in the case of the repeated BIS data sorted based on the cell lines, the compared measurements are well correlated (Supplementary Fig. S3). For the 231 and MCF7 cell lines, the correlation coefficient values are between 0.98 and 1, however for MSC cells, they are generally even higher, they are varying between 0.99 and 1. The typical correlation coefficient value for the cell lines is also around 0.99.

Afterwards, the model fitting results and the extracted Cole–Cole parameters are presented. The “worst-fitted” model, is characterized with the value of $R^2 = 0.9959$, while the best value is characterized with $R^2 = 0.9994$.

The properties of the extracted model parameters are summarized in the Supplementary Table S3 online. In case of the extracted model parameters, the CV values are generally in the range of 5–20 %. This is higher than the maximum variance of 5 % observed in the saline validation experiments.

**Figure 7:**
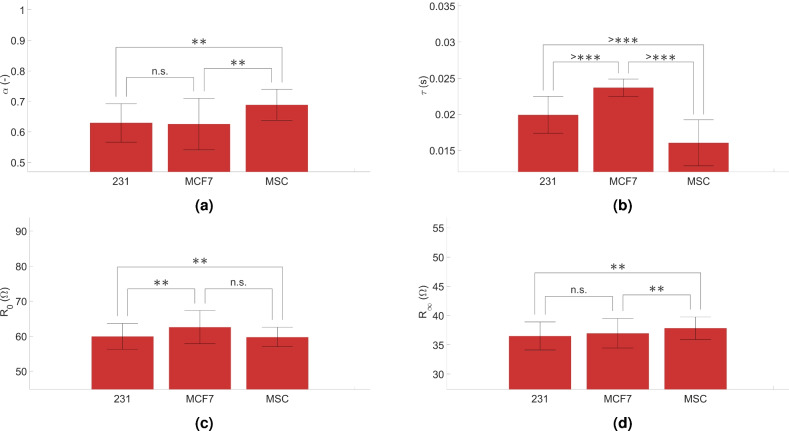
Comparison of $\alpha$, $\tau$, $R_0$ and $R_{\infty }$ values for each cell type using a two-sided Kolmogorov–Smirnov test (figures in case of the (**a**) $\alpha$, (**b**) $\tau$, (**c**) $R_0$ and (**d**) $R_{\infty }$).

The model parameter based comparison of cell lines is realized using the two-sided Kolmogorov–Smirnov test. The p-values are summarized in Supplementary Table S4 online and illustrated with bar charts grouped by Cole–Cole parameters in Fig. 7.

In the first case, the cell lines are compared using the $\alpha$ parameter extracted from the corresponding BIS data. The results are introduced in Fig. 7a. The results of significance tests obtained from the $\alpha$ parameter groups are the following. As it can be observed in Fig. 7a, the difference between 231 and MCF7 cell types is considered as not significant $R_{\infty }$, however, there is a significant difference obtained between the 231 vs MSC (p = 0.0082) and MCF7 vs MSC (p = 0.0082) parameter set comparisons. The column heights and the standard deviations also indicate these results, although the mean values and standard deviations for 231 and MCF7 cells are visibly similar. In the case of the MSC cell line, it can be seen that the mean value is significantly different from the other cells and the standard deviation lines are less overlapped. In the next step, the significance test results of the $\tau$ parameter groups are demonstrated in Fig. 7b.

Based on Fig. 7b, it can be concluded, that the $\tau$ parameters of all three cell types differ significantly (the p-values are near to zero in all of the cases). The significance level of the difference between the groups is 0.0001, as it is shown in Fig. 7b. There is also a visibly significant difference in the column heights and a small overlap in the bars representing the standard deviations. The significance test for the $R_0$ parameters lead to the results shown in Fig. 7c.

In Fig. 7c, comparing the $R_0$ values by cell types, it can be seen that the column heights, i.e. the mean values, are visibly close to each other and the variances overlap more than, for example, in case of the $\tau$ data (in Fig. 7b). Based on the p-values from the Fig. 7c, the difference between MCF7 and MSC is not considered as significant (the p-values are approximately equal to 0.05), however, it is noticed, that the 231 vs MCF7 and 231 vs MSC data sets are significantly different (the p-values are less than 0.01).

The comparison of $R_{\infty }$ data shows that the mean values are relatively close, and the standard deviation bands are overlapped. From the p-values of the $R_{\infty }$ mean values in Fig. 7d it can be seen, that the difference between the cell lines, based on the statistical analysis for $R_{\infty }$ data sets, for 231 and MCF7 cell types is not significant (the p-values are approximately equal to 0.05), however in the case of 231 vs MSC and MCF7 vs MSC, in Fig. 7d significant differences can be measured in $R_{\infty }$ parameter (the p-values are less than 0.01).

### Discussion

Based on the results of the cell viability assays, neither the graphene electrode surface, nor the application of the excitation current injected during the measurements induced any damage in the examined 231, MCF7 and MSC cell cultures (Section “Cell viability assay”). All measurements were performed with a voltage-generator excitation (100 mV peak-to-peak), which (according to the Supplementary Fig. S5) resulted in an average maximum current of about 110 $\mu A$ during the whole experiment. These results of viability tests (described in Section “Viability results”) suggest, that BIS plates are suitable for cell tracking, since neither the attachment to the plate, nor the BIS measurements affect the cell viability, proliferation, or morphology.

Based on the validation measurements (in Section “Physiological saline measurements”) performed on a series of physiological saline solutions (Fig. 2b), the obtained results (described in Section “Plate validation results”) are well correlated and they are visibly separable in the case of different saline concentrations. As it was described in Section “Plate validation results”, the BIS data, in each saline concentration group, follow the normal distribution regardless of the time of fabrication and validation of the plates. In the case of the variance of each concentration group, the CV values are decreasing as the concentration increases. The variance of the 100% solution is calculated around 3.26%.

Regarding the medium control plates in Fig. 5 clearly shows, that although the 100% saline solution and medium curves, measured in the same Petri dish did not differ significantly, there was a significant difference between the two spectra and the cell BIS data. Based on of these results, the effect of the coating and the medium is negligible in in vitro biological experiments. Thus, the BIS information, measured with the proprietary technique, is considered as a consequence of purely biological changes.

In the case of the medium control plates, comparing the results of the two (Supplementary Figure S2) and four-electrode measurements (Fig. 5), it is observed that the two-electrode measurements have extremely high variance on the cell culture and are not separated from the spectra of the saline and/or medium. The reason for this phenomenon is the instability of the electrical double layer and cell contact on the excitation electrodes (no special treatment was applied to the electrodes in order to stabilize that). However, a significant reduction in variance and stable measurement results were observed in the four-electrode measurements. However, in the four-electrode measurements a significant reduction in variance and stable measurement results were observed due to the minimization of the influence of the electric double layer. Further, it can be concluded from the spectra of the 100% saline solutions (Fig. 5), that the $Z_S$ impedance (in Fig. 1) is a frequency dependent circuit element (compared to the solution resistance in the simplified Randles cell^40^), which implies that the four-electrode method is unable to eliminate, but rather to suppress, the effect of the electric double layer^52^.

In the case of the Cole–Cole model parameters of the cell line BIS data magnitude, the goodness-of-fit remained above $R^2 = 0.99$ even for the “worst fit” suggesting, that the extracted model parameters are a good representation of the measured data. The statistical evaluation of the Cole–Cole parameters shows (Supplementary Table S3), that the stability of the method is most indicated by the fact that the CV values for the same parameters (regardless of the cell line) are of approximately the same magnitude order. In fact, it is remarkable that the CV values of the $R_0$ and $R_{\infty }$ parameters are very similar in all cases (Supplementary Table S3). Based on the results in Fig. 7, the comparison of the $\alpha$ and $R_{\infty }$ parameters didn’t show significant difference in the case of the of two tumor cell lines (231 and MCF-7). In contrast, the same parameters of MSC cells significantly differ from tumor cell lines. All three cell lines showed significant differences in the $\tau$ parameter compared to each other. Moreover, comparing the $R_0$ parameters, MCF7 and MSC lines were not statistically different, however, both cell cultures showed significant differences compared to the 231 cell line.

To this end, it is crucial to examine how the variance of the measurement results changes with increasing the complexity of the measurement assembly. Considering the results presented by Vizvari et al.^46^ as a baseline where the variance of the investigated prototype changed in the ppm range, the first extension of the prototype is the in vitro assay and the addition of a 100 % physiological salt solution, where electrodes and electrochemical phenomena complicate the measurements. The findings here confirm that the range of variance of the data is of the order of a few percent (Supplementary Table S2). The measurement setup reaches its maximum complexity when data are collected using the cell culture, as the biology adds a higher level of complexity in addition to the electrochemical effects. The results show that the variance of the data, even in this case, did not increase significantly. According to the Supplementary Table S3, the CV values of the parameters $R_0$ and $R_{\infty }$ (regardless of the cell type) are similar to those for physiological saline. In contrast, the variance of the $\tau$ and $\alpha$ parameters almost doubled as a consequence of the addition of cell culture. Determining whether this increase is due to technical or biological effects will be part of a forthcoming study.

## Conclusion

In this work, the aim is to confirm the non-destructiveness, reproducibility, and precision of a self-developed, in vitro BIS method. As the conclusion of the conducted tests to assess the quality of plate production and preparation, it has been determined that the innovative in vitro BIS method enables the comparison of BIS measurements, that are completely independent of each other in terms of both, space and time.

Based on these medium control results, it has been concluded, that the proposed BIS method is highly sensitive to live cells in the in vitro experiments. Moreover, it has been demonstrated, that the Cole–Cole parameters extracted from the high correlated BIS data, recorded for the identical cell line, show normal distribution regardless of the time when the plaques were produced and/or the experiments are performed. The research findings have also demonstrated that the four-electrode data collection and special evaluation method is capable of stabilizing the spectra measured on cell cultures and suppressing the instabilities of the electric double layer on the excitation electrodes. This is also suggested by the comparison of the two vs. four-electrode measurements and results of the statistic analysis of the Cole–Cole parameters. According to the comparative statistical analysis of the obtained Cole–Cole parameters, the introduced method is capable for distinguishing individual tumor cell lines on the basis of impedance spectra.

Now, the only limitation of the technology is the large cell and material requirement due to the large Petri dishes. Therefore, the short-term goal is to rationalize and reduce the size of the measuring plates in order to optimize the necessary cost requirements. Accordingly, a number of other electrode materials (gold, platinum, transparent ITO) will be investigated.

The current phase of the research encourages to continue the research based on the achieved results in the manner of the characterization of different tumor and non-tumor cell lines using the developed method with Cole–Cole model. In future, the developed technology will allow the establishment of a BIS database of cells under investigation, and then the identification of cells based on the model parameters will be available. In addition, the research is intended to be extended with the time domain studies too. Therefore, a future plan is to use technology in order to study biological processes such as cell number changes, viability, cell death, morphological changes and responses to different treatments, that can be associated with different cell-electrode mathematical models.

### Supplementary Information

Supplementary Information.

## Data Availability

The datasets used and/or analyzed during the current study available from the corresponding author on reasonable request.
