# Supplementary material for: Reproducibility analysis of bioimpedance-based self-developed live cell assays
Source: Sci Rep. 2024 Jul 16;14:16380. doi: 10.1038/s41598-024-67061-2 (PMC11252348; doi:10.1038/s41598-024-67061-2)
Supplement: Supplementary file 1 — Supplementary Information. [file 41598_2024_67061_MOESM1_ESM.pdf]

Supplementary information for the manuscript:

Reproducibility Analysis of Bioimpedance-Based Self-Developed Live Cell Assays

Vizvari et al.

S1 Supplementary Methods

S1.1 Self-Produced Graphene Plates

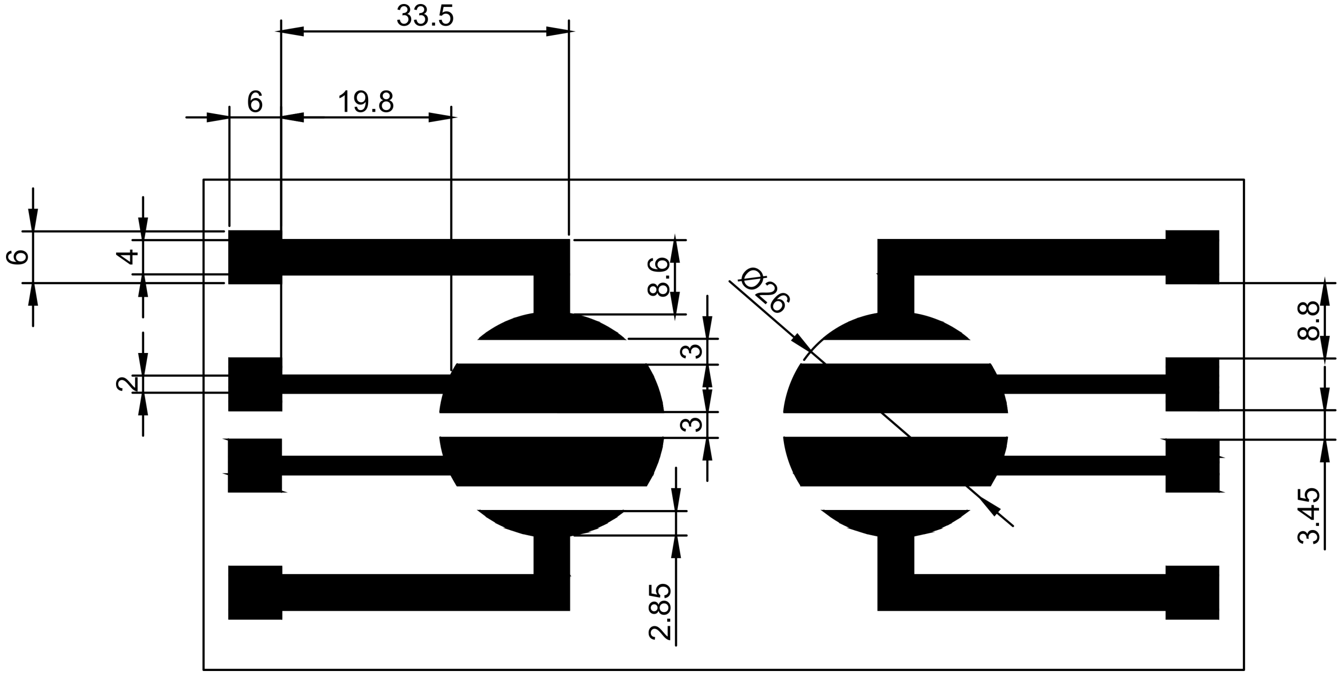

Supplementary Figure S1. The blueprint of the novel graphene-based BIS plate (all dimensions are in millimetres)

S1.2 The BIS measurement technique

Based on the measurement principles presented in the main text of the paper, the spectra (Z_{2el}) corresponding to the two electrode measurement methods can be calculated at each frequency point, using the potential values u_1, and u_4, according to the following formula:

$$Z_{2el} = R_{ref}\frac{u_1 - u_4}{u_4} = R_{ref}\left(\frac{u_1}{u_4} - 1\right) \tag{1}$$

S2 Supplementary Figure

S2.1 Medium control plates and cell line BIS results

The Z_{2el} spectra for 100% saline, medium controll and cell lines are as follows:

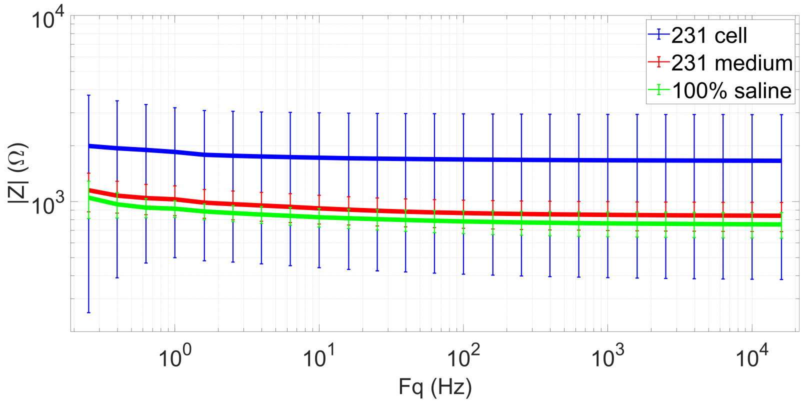

(a)

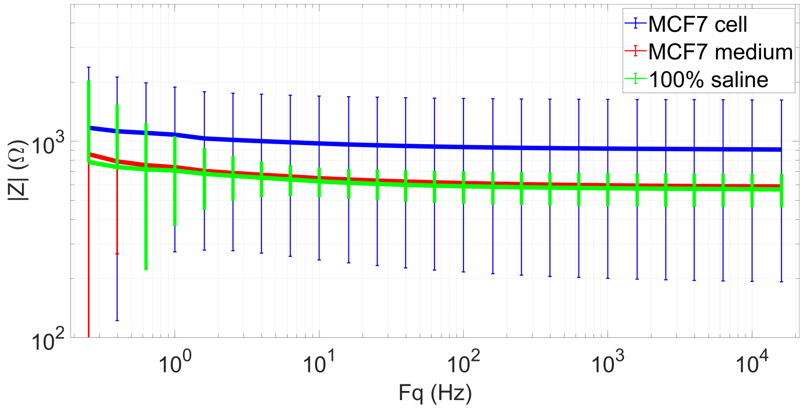

(b)

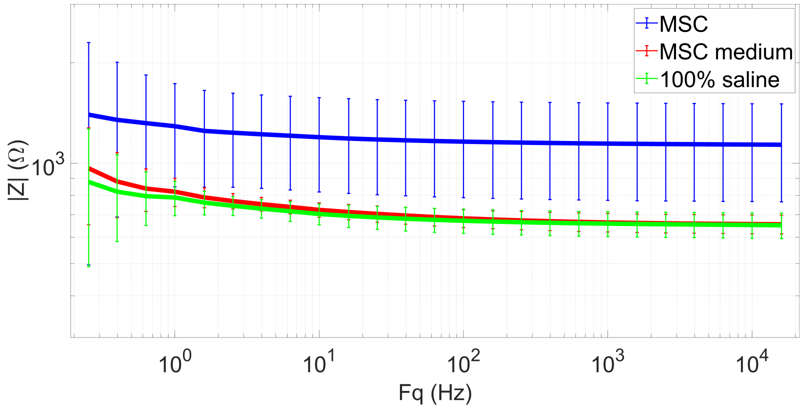

(c)

Supplementary Figure S2. Comparison of the 100 % saline, medium controll and the corresponding in vitro BIS data recorded using two electrode method in the case of 231 **(a)**, MCF7 **(b)** and MSC **(c)** cell lines (100% solutions and medium control results are only considered in the same Petri dishes)

S2.2 Cell culture BIS results

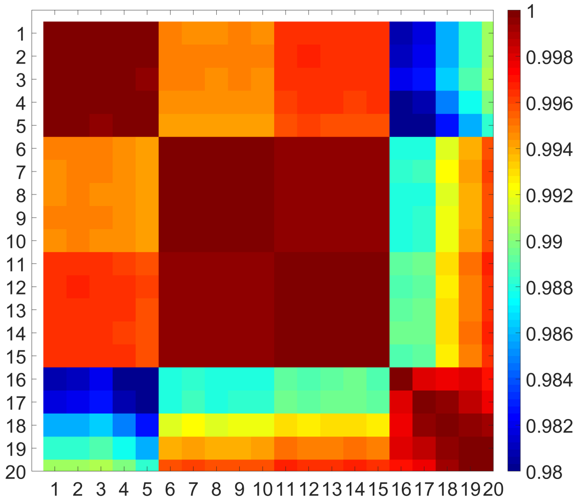

(a)

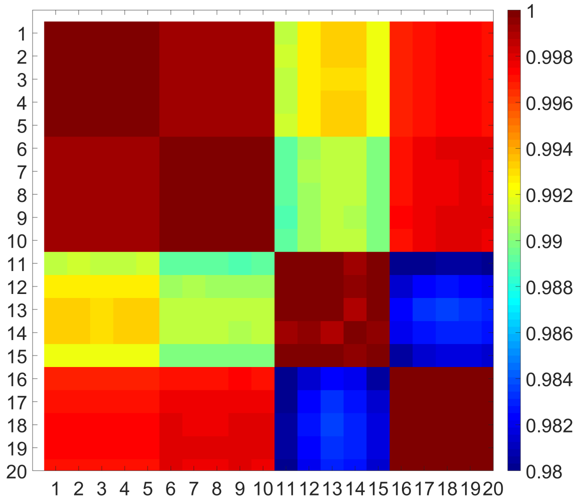

(b)

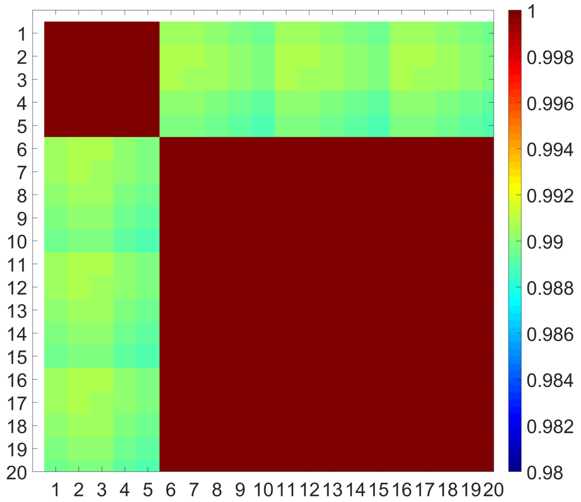

(c)

Supplementary Figure S3. Correlation matrices of BIS data measured on each cell culture calculated using Pearson correlation

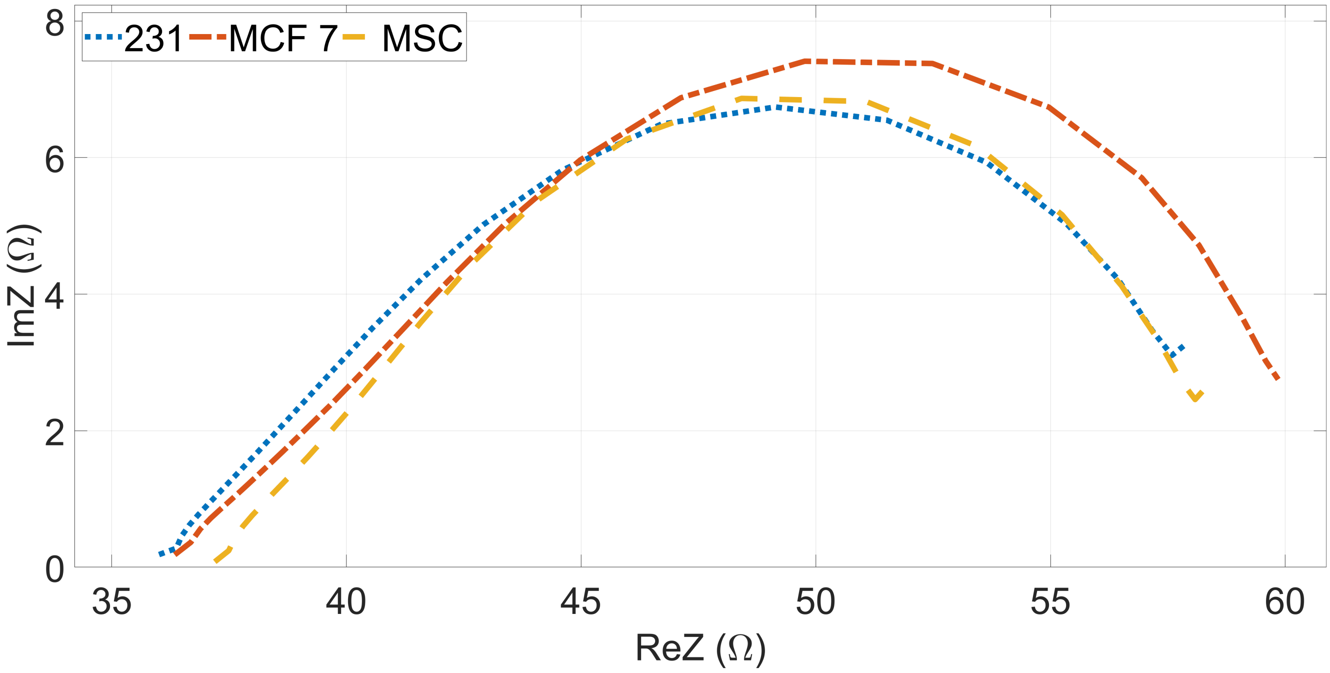

Supplementary Figure S4. The Nyquist plot of BIS data averages recorded on the cell culture

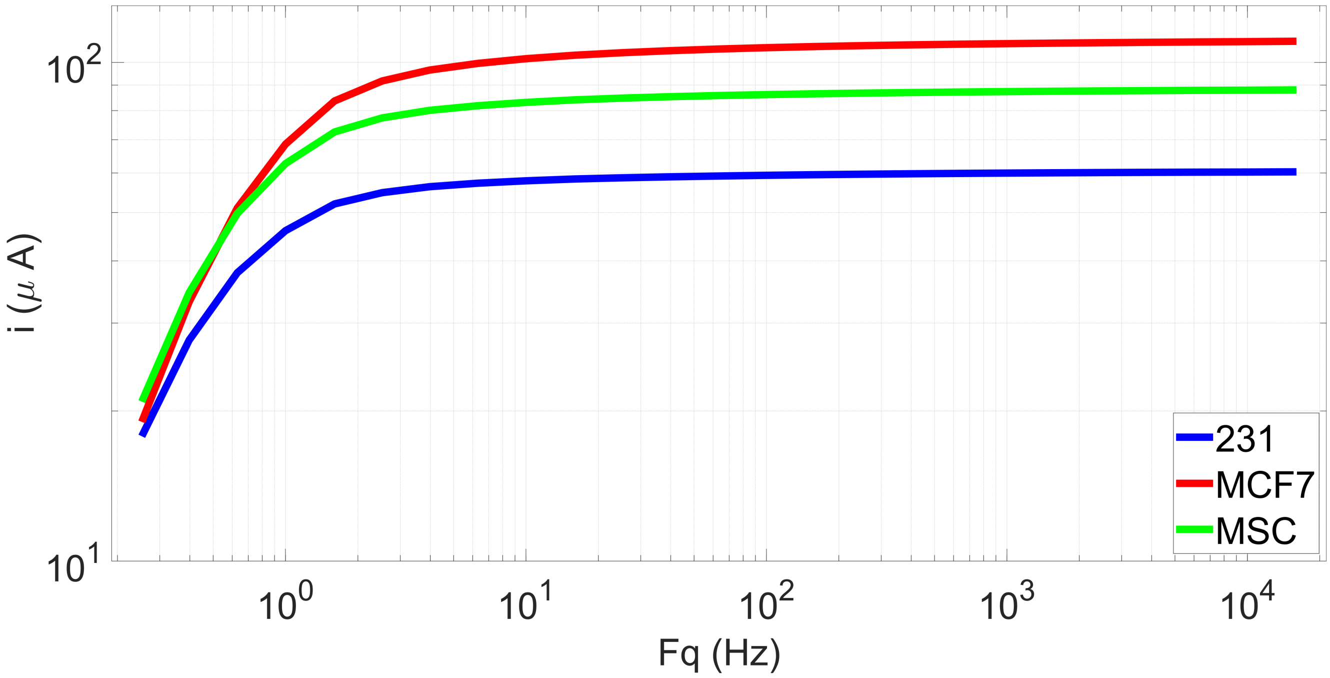

Supplementary Figure S5. The average currents for each cell culture as a function of frequency (calculated from the averages of the two electrode spectra and the 100 mV peak-to-peak excitation)

S3 Supplementary Tables

S3.1 Viability Tests Statistical Results

Cell line	time point	p-value	decision
231	24 h	0.6030	true
	48 h	0.9575	true
	72 h	0.9575	true
MCF7	24 h	0.6030	true
	48 h	0.6030	true
	72 h	0.9575	true
MSC	24 h	0.6030	true
	48 h	0.6030	true
	72 h	0.9575	true

Supplementary Table S1. The results of the two sided Kolgomorov-Smirnov significance test in the case of all the three cell lines in all time points (null hypothesis: the data in vectors are from the same continuous distribution)

S3.2 Plate Validation Statistical Results

Conc.	p-value	decision	CV (%)
0%	0.1264	true	67.5293
25%	0.0543	true	9.3897
50%	0.8294	true	2.4924
75%	0.7740	true	3.5335
100%	0.9677	true	3.2683

Supplementary Table S2. Results of the one-sided Kolmogorov-Smirnov normality test for impedance data at 160 Hz sorted by concentration (null hypothesis: data are from standard normal distribution)

S3.3 Cell culture Cole – Cole parameters

231 cell line					
CC param.	p-value	decision	Avg. val.	Std. val.	CV (%)
α ($-$)	0.0138	true	0.6292	0.0630	10.0191
τ (s)	0.4934	true	0.0199	0.0026	12.8985
R_0 (Ω)	0.0115	true	59.9497	3.6580	6.1018
R_∞ (Ω)	0.0699	true	36.5111	2.3739	6.5018
**MCF7 cell line**					
CC param.	p-value	decision	Avg. val.	Std. val.	CV (%)
α ($-$)	0.1999	true	0.6253	0.0843	13.4890
τ (s)	0.1998	true	0.0237	0.0012	5.1127
R_0 (Ω)	0.0973	true	62.5673	4.6877	7.4923
R_∞ (Ω)	0.0579	true	36.968	2.5201	6.8171
**MSC cell line**					
CC param.	p-value	decision	Avg. val.	Std. val.	CV (%)
α ($-$)	0.1537	true	0.6884	0.0508	7.3740
τ (s)	0.0382	true	0.0160	0.0032	19.7433
R_0 (Ω)	0.0151	true	59.7611	2.7735	4.6409
R_∞ (Ω)	0.3385	true	37.8351	1.9134	5.0571

Supplementary Table S3. Results of the one-sided Kolmogorov-Smirnov normality test (null hypothesis: data are from standard normal distribution), CV values, mean and standard deviation for Cole-Cole parameter sets

	231	MCF7	MSC
231	1	0.4973	0.0082
MCF7	0.4973	1	0.0082
MSC	0.0082	0.0082	1

(a)

	231	MCF7	MSC
231	1	$0.8418 \cdot 10^{-5}$	$0.8418 \cdot 10^{-5}$
MCF7	$0.8418 \cdot 10^{-5}$	1	$0.0001 \cdot 10^{-5}$
MSC	$0.8418 \cdot 10^{-5}$	$0.0001 \cdot 10^{-5}$	1

(b)

	231	MCF7	MSC
231	1	0.0082	0.0082
MCF7	0.0082	1	0.4973
MSC	0.0082	0.4973	1

(c)

	231	MCF7	MSC
231	1	0.4973	0.0082
MCF7	0.4973	1	0.0082
MSC	0.0082	0.0082	1

(d)

Supplementary Table S4. The p-values calculated using the two-sided Kolmogorov-Smirnov test in case of the cell culture Cole - Cole parameters **(a)** α, **(b)** τ, **(c)** R_0 and **(d)** R_∞
